# Nutritional ecology of a prototypical generalist predator, the red fox (*Vulpes* *vulpes*)

**DOI:** 10.1038/s41598-024-58711-6

**Published:** 2024-04-04

**Authors:** A. Balestrieri, S. Gigliotti, R. Caniglia, E. Velli, F. Zambuto, E. De Giorgi, N. Mucci, P. Tremolada, A. Gazzola

**Affiliations:** 1 Dipartimento di Scienze e Politiche Ambientali, Università di Milano, via Celoria 26, 20133 Milan, Italy; 2 Dipartimento di Biologia, Università di Padova, via Ugo Bassi 58/B, 35131 Padua, Italy; 3 Dipartimento di Scienze della Terra e dell’Ambiente, Università di Pavia, 27100 Pavia, Italy; 4 Area per la Genetica della Conservazione, Istituto Superiore per la Protezione e la Ricerca Ambientale (ISPRA), via Ca’ Fornacetta 9, Ozzano Emilia, 40064 Bologna, Italy; 5 IRCCS Ospedale Galeazzi-Sant’Ambrogio, via C. Belgioioso 173, 20161 Milano, Italy

**Keywords:** Animal behaviour, Animal physiology, Ecology

## Abstract

Generalist species, which exploit a wide range of food resources, are expected to be able to combine available resources as to attain their specific macronutrient ratio (percentage of caloric intake of protein, lipids and carbohydrates). Among mammalian predators, the red fox *Vulpes* *vulpes* is a widespread, opportunistic forager: its diet has been largely studied, outlining wide variation according to geographic and climatic factors. We aimed to check if, throughout the species’ European range, diets vary widely in macronutrient composition or foxes can combine complementary foods to gain the same nutrient intake. First, we assessed fox’s intake target in the framework of nutritional geometry. Secondly, we aimed to highlight the effects of unbalanced diets on fox density, which was assumed as a proxy for Darwinian fitness, as assessed in five areas of the western Italian Alps. Unexpectedly, the target macronutrient ratio of the fox (52.4% protein-, 38.7% lipid- and 8.9% carbohydrate energy) was consistent with that of hypercarnivores, such as wolves and felids, except for carbohydrate intakes in urban and rural habitats. The inverse relation between density and the deviation of observed macronutrient ratios from the intake target suggests that fox capability of surviving in a wide range of habitats may not be exempt from fitness costs and that nutrient availability should be regarded among the biotic factors affecting animal abundance and distribution.

## Introduction

Variation in food availability is a major factor affecting breeding densities and reproduction success, with ultimate consequences on population dynamics^1^. Availability of food resources is expected to act as a limiting factor more for specialist predators, which use one or few resources, than for generalist predators, which opportunistically exploit the most abundant and easily accessible food resources available at any one time and locality^2^.

Whether the two feeding tactics are equally profitable is still debated^3,4^, especially for terrestrial predators^5^. According to optimal foraging models, trophic niche breadth should depend on the diversity and abundance of available prey, and net energy gain^6^, that is the ratio between the caloric value of each potentially available prey and the amount of energy spent for finding, pursuing, killing and consuming it.

Notwithstanding, in the last two decades, a growing body of evidence has outlined that foraging is not exclusively driven by energy acquisition and many species tend to regulate the macronutrient composition (percentage of caloric intake of protein, lipids and carbohydrates, P:L:C) of their diet to a target macronutrient ratio (“intake target”)^7–9^. As the macronutrient composition of the diet has been demonstrated to affect many fundamental fitness-related traits, including growth^7,10^, fecundity^11^ and lifespan^12,13^, we may expect that, for any species, suitable habitats are those which offer food resources capable of satisfying the nutritional requirements of a number of individuals in all the phases of their life cycle.

Specialist predators possess morphological and behavioural adaptations which are supposed to increase their foraging efficiency^14^ and feed on foods relatively invariant in their nutrient composition, which coincides with the predator’s intake targets. In contrast, generalists must be capable of shifting between resources before the opportunity occurs, which implies preexisting behavioural and physiological adaptations^15^, and need to combine several nutritionally complementary foods to achieve their intake target.

The few available studies^16^ suggest that, based on their tolerance towards carbohydrates, mammalian predators can be aligned along a carnivore–omnivore continuum, ranging from obligate carnivores, such as wolves (*Canis* *lupus*)^17^ to poorly specialized ursids^18^. The ability of using fat or carbohydrates as sources of non‐protein energy may be expected to be a physiological prerequisite for generalist predators, allowing them to rely on a wide variety of food resources^19^.

Geographic and seasonal variation in the composition of generalist predators’ diets makes it difficult to compare the diet of populations of widespread species. However, using nutritional geometry Gazzola and Balestrieri^20^ have recently demonstrated that using a wide variety of food resources does not imply as much variation in the macronutrient composition of diets: although using a wide range of fruit and small mammals, widespread carnivores such as martens (*Martes* *martes* and *Martes* *foina*) can be considered macronutrient specialists (i.e. the macronutrient compositions of the diets of different populations are similar^21^).

Among carnivores, the red fox (*Vulpes* *vulpes*) is considered a prototypical generalist predator: its feeding habits vary widely spatially, temporally and in response to human influence, reflecting the biogeographical patterns of distribution and abundance of food resources^22–24^. Records of local specialization, due to the disproportionate profitability of anthropogenic resources, reflect the highly opportunistic behaviour of this species^25^.

This dietary flexibility allows foxes to occur in a wide variety of habitats, from sea level up to 4500 m, including several cities^26^. Its geographical range is the widest of any member of the order Carnivora (ca. 70 million km^2^), including most of the Northern Hemisphere, from the Arctic Circle to northern Africa, and Australia^27^, where it was introduced in the 1870s^28^.

Such a wide distribution rises an interesting question, that is whether different populations persist on diets that vary widely in macronutrient composition or are capable of using complementary foods to gain the same nutrient intake throughout the species’ range.

The first nutritional strategy has been reported for the wild boar (*Sus* *scrofa*), which is a dietary generalist and tolerates a wide range of macronutrient ratios across its whole range, particularly in terms of proportion of energy from protein^29^. In contrast, mustelids, such as martens (*Martes* spp.) and the Eurasian badger (*Meles* *meles*), tend to keep constant the percent protein energy, while showing a gradient of tolerance towards carbohydrates^16,20^.

Laboratory experiments on *Drosophila* *melanogaster*^11^ and mice^12^ suggest that unbalanced diets may have profound effects on life span and reproduction. While the broad fundamental macronutrient niche of wild boars has been suggested to enhance their invasion success, increasing the reproductive output of sows^29^, we still do not know whether an excess of carbohydrates may affect the individual fitness of free-ranging carnivores.

To assess the macronutrient niche of the red fox, we applied right-angle mixture triangles (RMT)^21,30^, in the framework of nutritional geometry. Data were extracted from published reports following the approach proposed by Remonti et al.^19,31^. Based on the wide variety of foods used by foxes, we expected a wide degree of inter-population variation in the percent energy provided by carbohydrates, as so as the recording of clusters of unbalanced diets.

Secondly, we made an attempt to highlight the effects of unbalanced diets on fox density, which was assumed as a proxy for population fitness (i.e.: the capacity to survive and reproduce)^32,33^. As diet is only one of several factors that may affect population density, samplings were carried out in five areas belonging to the same biogeographical region, in a radius of ca. 30 km (western Italian Alps). Variation in rainfall and vegetation cover were expected to affect food availability to foxes. We aimed to assess both the yearly diet of each population and their correspondent density, calculated through a faecal DNA-based genetic census. We expected macronutrient ratios to affect individual fitness, and, therefore, populations showing nutritional balances close to the intake target to achieve higher densities than those with unbalanced diets.

## Results

### Estimation of the intake target

Protein energy ranged between 36.8 and 71.0%, lipid energy from 25.7 to 51.4%, while carbohydrate energy made up between 0.1 and 29.9%. The target macronutrient ratio of the fox (mean ± SE) was assessed as 52.4 ± 1.7% protein energy, 38.7 ± 1.0% lipid energy and 8.9 ± 1.6% carbohydrate energy (Table 1).

**Table 1:** Red fox (*Vulpes* *vulpes*) macronutrient (Protein, Lipids, Carbohydrates) intakes as assessed by the analysis of the 30 selected diet studies (references as for Fig. 4).

N	Sample size	P	L	C	Habitat	Lat	Long
1	121	43.84	39.59	16.56	Forest	45.44	7.19
	117	36.82	37.61	25.57	Urban		
2	115	66.57	33.06	0.37	Arable	45.10	8.38
3	133	41.58	29.57	28.85	Forest	46.26	11.45
4	78	37.97	35.65	26.37	Mixed	45.25	8.53
	114	49.67	44.16	6.17	Mixed	45.16	9.37
5	1139	59.45	38.49	2.05	Mixed	51.48	19.53
6	340	61.60	35.44	2.96	Mixed	43.31	11.08
7	663	40.07	44.55	15.38	Arable	42.76	11.33
8	393	68.32	31.55	0.13	Arable	50.45	− 2.26
9	749	37.46	32.58	29.95	Arable	51.27	− 2.35
10	922	62.23	30.68	7.09	Forest	45.59	7.08
11	234	57.21	41.60	1.19	Forest	55.02	29.02
12	264	44.10	49.76	6.15	Forest	41.41	13.50
13	223	51.11	43.05	5.84	Mixed	44.51	8.75
14	189	41.69	39.57	18.74	Forest	44.30	8.92
15	178	53.33	41.36	5.31	Forest	37.08	3.23
	678	61.37	33.15	5.48	Forest	41.03	4.53
16	933	55.66	42.72	1.62	Forest	53.00	27.50
	593	63.42	33.20	3.37	Forest		
17	767	54.17	44.53	1.30	Mixed	55.51	22.50
18	1010	44.49	51.43	4.08	Forest	46.41	17.45
19	256	43.03	31.19	25.77	Urban	51.00	13.00
20	190	51.11	42.71	6.17	Mixed	39.00	22.00
21	144	56.15	43.12	0.73	Forest	49.26	20.00
22	570	70.96	25.74	3.30	Forest	59.40	15.30
23	148	42.35	45.76	11.89	Mixed	55.16	25.33
24	102	57.55	40.37	2.08	Mixed	27.25	− 8.17
25	433	50.88	45.00	4.12	Arable	51.34	17.40
26	1022	59.02	36.55	4.43	Arable	51.34	17.40
27	320	69.19	29.05	1.75	Forest	37.16	6.43
28	1939	37.64	38.71	23.66	Urban	51.45	− 1.15
29	268	44.07	46.06	9.87	Arable	45.51	17.56
30	159	57.59	41.38	1.03	Arable	48.46	2.17
	169	61.75	32.46	5.78	Forest		
	220	56.30	42.10	1.59	Urban		
Mean		52.35	38.75	8.89			
SE		1.84	1.13	1.71			

**Figure 1:**
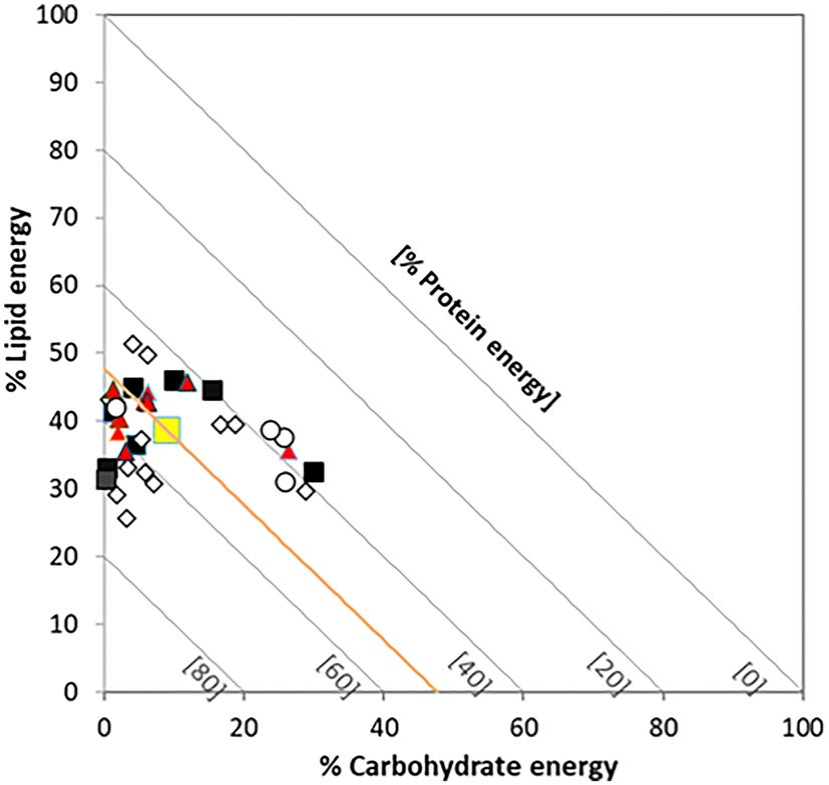
Right-angled mixture triangle showing the macronutrient ratios of the 30 selected diet studies (squares: arable land, dots: urban habitats, triangles: mixed habitats, diamonds: forests). The yellow square marks the intake target (mean macronutrient ratio). The coordinate for the implicit variable is read as the difference between 100% and the value at which the diagonal with a slope of − 1 that passes through the point identified by the primary coordinates intersects the I‐axis.

Overall diets clustered into three groups: ‘average’ (%P = 50 ± 5), mainly from mixed habitats, ‘low P‐high C’ (%P = 40 ± 5), including mostly diets of urban and cultivated areas, and ‘high P’ (P% > 60%) diets from mixed and forested habitats (Fig. 1). On average, carbohydrate energy tended to increase along the natural-to-urban habitat gradient, while lipid energy was the highest in mixed habitats (Table 2). Higher than average carbohydrate energies were recorded in mountainous, forest areas of northern Italy (1, 3 and 14 in Table 1), where fruit accounted for 30–50% of the diet (%mV). High protein intakes were related to the consumption of lagomorphs in both cultivated (2, 8) and low-altitude forest areas (27, 30), wild deer in mixed habitats (6) and ungulates (10: NW Italian Alps, 22: Sweden) or small mammals (16: northern Belarus) in woodland.

**Table 2:** Inter-habitat variation of the red fox (*Vulpes* *vulpes*) macronutrient intake (± SE) along the natural-to-urban gradient, based on the 30 selected diet studies.

Habitat	% protein	% lipids	% carbohydrates
Forest	55.0 ± 2.7	37.5 ± 2.1	7.5 ± 2.2
Mixed	51.7 ± 2.6	41.1 ± 1.3	7.2 ± 2.6
Arable land	53.0 ± 4.1	38.8 ± 2.1	8.2 ± 3.6
Urban	43.4 ± 4.5	37.4 ± 2.3	19.2 ± 5.9

### Fox diet in Alpine habitats

Overall, 391 km of transects were surveyed (mean ± SE: 78.2 ± 11.5 km per area; min–max: 56.0–116.5), yielding 615 faecal samples (mean ± SE: 126.7 ± 11.6 samples per area; min–max: 114–144). The analysis of fox diet showed differences in the relative importance of the major food items in the five study areas. Mice (*Apodemus* spp.) were the most frequent prey in all the three western areas, while voles (mostly *Myodes* *glareolus*) prevailed in Piedmont. In terms of volume, the highest values were achieved by ungulates in all study areas in Aosta Valley, while in two eastern sampling sites voles dominated also in terms of volume. As expected, fruits were less frequently eaten in winter while insects were most preyed on in summer. Ungulates, mostly eaten as carrions, were used in winter-spring. The frequency of occurrence of most major food items showed significant variation among areas (Table SI2), nonetheless, small rodents formed the bulk of fox diet in all study areas (Fig. SI1).

Protein energy ranged between 46.6 and 68.3%, lipid energy from 28.9 to 51.7%, while carbohydrate energy made up between 0.7 and 8.15% (Table 3). Overall, seasonal variation in the macronutrient ratios provided by diet was higher in the Aostan valleys than in Piedmont areas (Fig. SI2).

**Table 3:** Macronutrient ratios in the diet of the red fox (*Vulpes* *vulpes*) in the five study areas of the western Italian Alps (N: sample size).

Study area	N	% protein	% lipids	% carbohydrates
Nomenon	131	68.27	28.86	2.87
S. Barthelemy	117	47.71	44.14	8.15
Chalamy	125	56.29	40.70	3.02
Cervo	122	50.57	48.72	0.71
Elvo	120	46.56	51.70	1.74

The macronutrient intake of Saint-Barthélemy valley was the closest to the intake target, while the highest percent deviation of carbohydrate energy from the target were recorded for the two eastern areas (Table 4).

**Table 4:** Percent deviation from the intake target assessed for the five red fox (*Vulpes* *vulpes*) diets in Alpine habitats.

Study area	% protein	% lipids	% carbohydrates	Mean
Nomenon	30.4	25.5	67.7	41.2
S. Barthelemy	8.9	13.9	8.3	10.4
Chalamy	7.5	5.0	66.1	26.2
Cervo	3.4	25.7	92.0	40.4
Elvo	11.1	33.4	80.4	41.6

### Fox numbers

Genotyping success ranged between 35.7% for the valley of the River Chalamy and 82.8% for Saint-Barthélemy’s (mean: 52.8%). Sixteen different genotypes were recorded in Saint-Barthélemy valley, 9 each in Elvo and Nomenon valleys, 8 in Cervo valley and 7 in Chalamy valley. The number of “captures” per individual varied between 1 and 5.

Applying CAPWIRE’s TIRM model, the largest population was assessed for Saint-Barthélemy, with 30 individuals (CI: 16–30), followed by Nomenon with 28 individuals (11–30), Elvo with 23 (9–30), Cervo with 19 (8–30) and Chalamy with 12 (7–19). The lowest density was recorded for the Chalamy population, 0.7 ind/km^2^, and the highest for that of the Nomenon valley, 2.4 ind/km^2^ (Elvo: 1.9; S. Barthelemy = 2.2; Cervo = 1.73 ind/km^2^).

**Figure 2:**
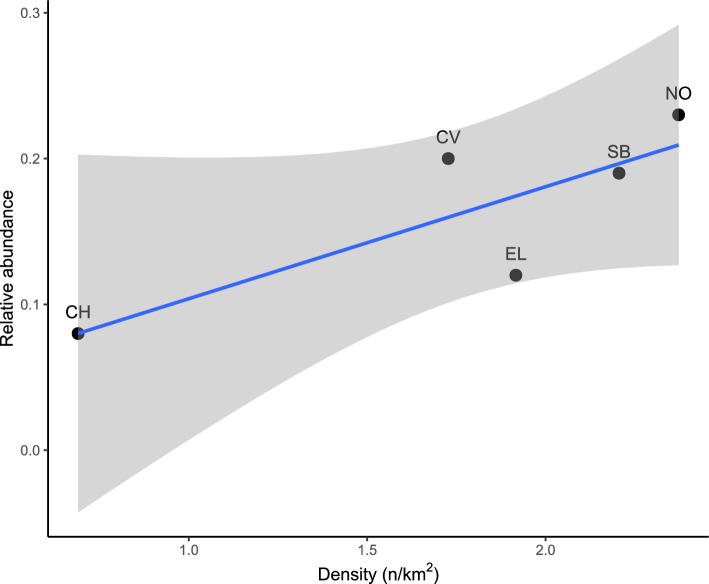
Relationship between red fox (*Vulpes* *vulpes*) relative abundance (RA = N of scats/100 m) and density in the five study areas (valleys of the rivers CH-Chalamy, CV-Cervo, EL-Elvo, SB-San Barthelemy and NO-Nomenon).

Fox relative abundance (RA) ranged between 0.08 and 0.24 faeces/100 m and tended to increase with density (P = 0.09, R^2^ = 0.67; Fig. 2).

**Figure 3:**
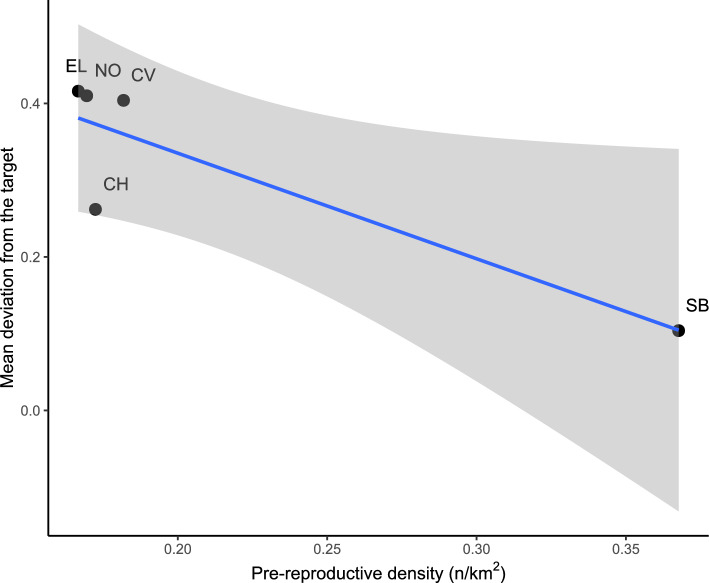
Relationship between pre-reproductive density and mean deviation of the macronutrient ratio from the intake target in the five study areas (valleys of the rivers CH-Chalamy, CV-Cervo, EL-Elvo, SB-San Barthelemy and NO-Nomenon).

Pre-reproductive density ranged between 0.17 and 0.37 ind/km^2^ (mean ± SE: 0.21 ± 0.04 ind/km^2^) and tended to decrease with increasing deviations of the macronutrient ratio from the target (P = 0.037, R^2^ = 0.78; Fig. 3).

## Discussion

The red fox occurs in a wide geographic range where it must cope with a diversity of environmental conditions and large variation in the availability of food resources. Its food habits have been widely studied, highlighting a great trophic diversity, which may be expected to result in an equally broad inter‐population variation in the macronutrient intake.

Notwithstanding, the analysis of available studies providing a volumetric or biomass estimate of the importance of the food resources used by foxes throughout its European range revealed that, on average, the protein requirements of the fox are typical of strict carnivores such as wolves (54%)^17^ or domestic and feral cats (52%)^34,35^. Respect to hypercarnivores, foxes seem to tolerate some carbohydrates in their diet, although their contribution was usually lower than expected based on their opportunistic food habits.

These results underpin the need for considering macronutrient ratios to draw an effective picture of generalist predators’ diets, because food diversity can conceal their actual nutritional requirements^21^.

As reported for badgers^31^, in urban and rural habitats macronutrient ratios differed the most from the target, particularly for percent carbohydrate energy. Carbohydrate intake is probably affected by the availability of anthropogenic food resources, given the opportunity of searching for food in garbage cans, compost piles and orchards^36^. While shortage in animal prey, particularly in summer, has been reported to affect survival and/or fecundity in another canid, the coyote (*Canis* *latrans*)^37^, no information is available, to the best of our knowledge, about the detrimental effects of carbohydrate overeating. Carbohydrates are generally considered noxious to carnivores, inducing sharp changes in intestinal metabolism and interfering with the digestion of protein and absorption of minerals^38^. Nonetheless, there is no evidence that a high consumption of fruit during summer could impair the reproduction of red foxes during the following spring^39^, suggesting that carbohydrates may be well tolerated by foxes, or even partially necessary for a balanced diet.

The reviewed dietary studies aimed to determine the relative importance of food items in the diet rather than the absolute amount of food consumed or their macronutrient composition. We acknowledge that assessing macronutrient ratios using such studies cannot but provide a rough estimate of the actual intake target of the fox. Nonetheless, the analysis of fox diet in the five Alpine areas, which was carried out by assessing the relative volume of each food category as carefully as possible, allowed to assess macronutrient ratios consistent with the general picture drawn through the literature review, yielding macronutrient ratios similar to those assessed for most fox populations living in forested areas throughout Europe.

In our study areas, fox diet was poor in fruit (average Vm% = 8.5) respect to previous studies carried out in the western Italian Alps (Vm% = 15–32%)^40–42^. Based on anecdotal information, in summer 2021 rodents were very abundant, because of a mast year for beech (*Fagus* *sylvatica*), suggesting that the recorded shift may depend on the higher-than-average availability of this food resource, as already recorded for martens in NW Piedmont^43^.

Average genotyping success (52.8%) was consistent with previous studies based on faecal DNA (e.g., 48%^44^, 58%^45^). Densities fell within the range reported for Italian fox populations (1–2.5 foxes/km^2^)^46^. Although the use of marking intensity as an index of relative abundance or for assessing habitat preferences has been long challenged^47,48^, the recorded relationship between density and the index of relative abundance suggests that marking intensity can be used as an effective index to compare fox abundances (see also Lanszki et al. about *Lutra* *lutra*^49^).

To investigate the effects of the macronutrient composition of fox diet on population fitness, we assessed winter densities, which were assumed to be less sensitive to variation in local conditions (e.g., number of cubs, percentage of barren females) than post-reproductive densities^50^. Mean values were consistent with those reported by Bartoń and Zalewski by reviewing 69 studies throughout Europe and Asia^51^.

While we are well aware that sample size is too low to draw sound conclusions, the inverse relation between density and the deviation of observed macronutrient ratios from the intake target suggests that the nutrient composition of available foods can drive fox abundance, affecting the chance of achieving diets able to satisfy its nutritional, i.e. physiological, requirements. Although density is only a rough proxy for fitness, our results are consistent with laboratory experiments, which demonstrated that generalists pay the cost of relying on unbalanced diets, suffering either high mortality rates and disease risk^52,53^ or low reproductive outputs^12^.

This result implies that although foxes can adapt to local and seasonal variations in food availability and then survive in a wide variety of habitats^24^, this capability may not be exempt from fitness costs. Nutrient availability should be considered, together with habitat productivity^54^, among the biotic factors affecting animal abundance and distribution.

## Conclusions

Following Machovsky-Capuska et al.^21^, by analysing the nutritional niche of a well-known generalist predator we demonstrated that the characterisation of dietary niches cannot disregard the nutritional composition of food resources. The red fox, although being capable of relying on foods largely varying in their nutrient composition, showed to “defend”^8^ the protein intake target typical of hypercarnivore mammals. Moreover, we provided some field-based evidence that not only food availability per se but also the macronutrient composition of foods may affect at least animals’ distribution, if not their life history traits.

## Materials and methods

### Assessment of the intake target

Following Remonti et al.^31^, we searched the available literature using the keywords: “diet,” “food habits”, “trophic niche,” “fox”, “*Vulpes*” and “macronutrients”. We found 73 papers and selected the studies based on the following criteria: (i) results had to be expressed as percent volume or biomass; (ii) the study lasted at least one year (4 seasons); (iii) the number of analysed samples had to be higher than 60. The last two criteria intended to select only those studies providing an effective picture of fox diet. Thirty studies met these criteria and were used to assess the intake target. As environmental conditions may imply different resource availability, based on the description of the study areas, the dataset was split in four main habitats: Urban, Arable, Mixed, and Forest habitats (Table 1).

**Figure 4:**
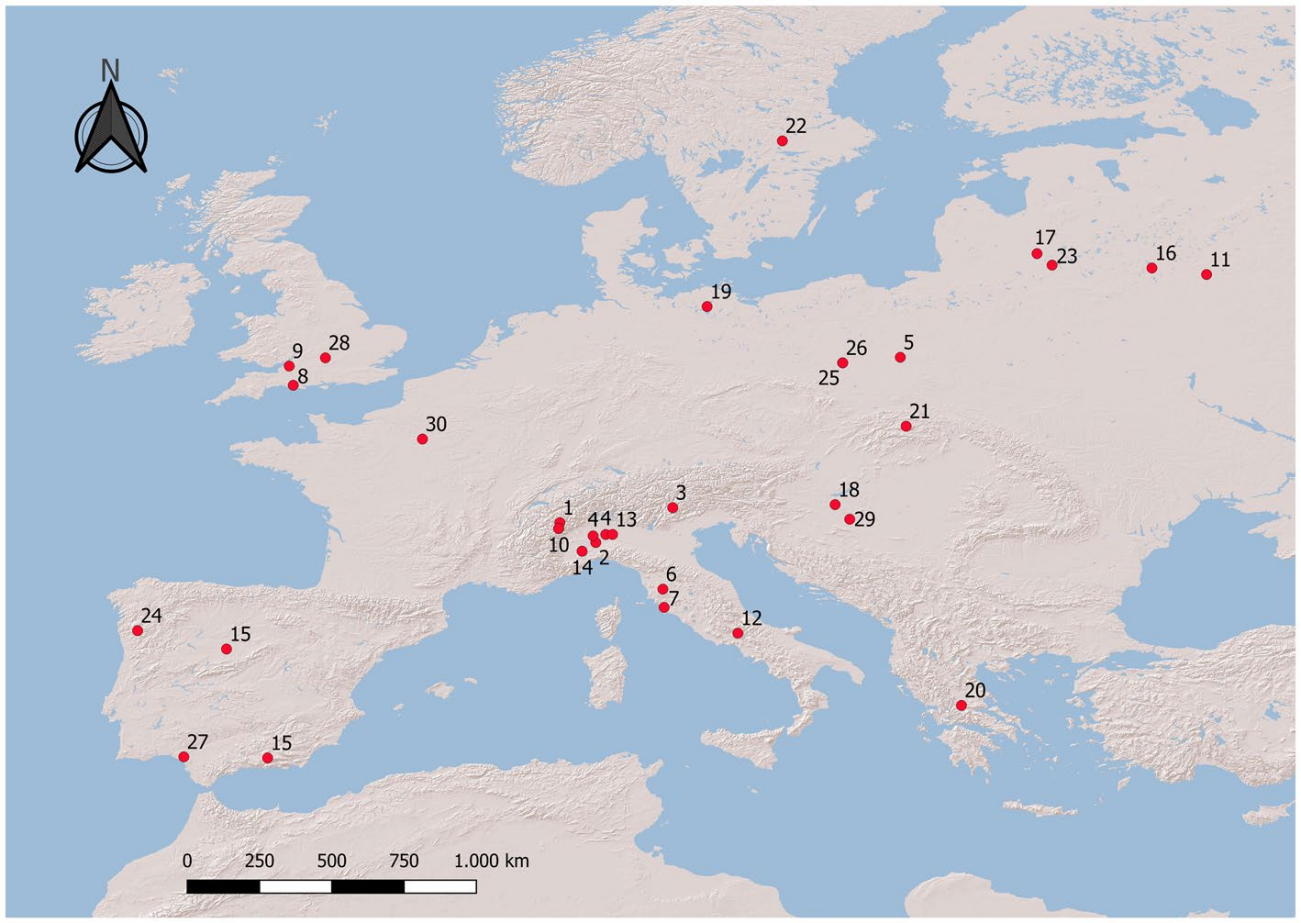
Location of the 30 selected studies (1^40^; 2^25^; 3^71^; 4^72^; 5^73^; 6^74^; 7^75^; 8^76^; 9^77^; 10^41^; 11^78^; 12^79^; 13^80^; 14^81^; 15^82^; 16^83^; 17^84^; 18^56^; 19^85^; 20^86^; 21^87^; 22^88^; 23^89^; 24^90^; 25^91^; 26^92^; 27^93^; 28^94^; 29^95^; 30^96^). Map created by authors using QGIS 3.14 (https://qgis.org/).

All the selected studies were conducted in Europe, ranging between 27° and 59° N in latitude and 8° W and 29° E in longitude (Fig. 4, Table SI1). The macronutrient ratio of each diet was assessed by multiplying the percent volume or biomass of every food item by the respective percentage of each macronutrient. To obtain, on a wet weight basis, the mean percentage of protein, lipids, and carbohydrates of the food items used by the red fox, we checked the available literature on the nutritional composition of food^17,31,32^ (Table 5). Undetermined items were assigned with the mean value calculated for the foods belonging to the same major group. Macronutrient energy ratios (MER) were calculated by multiplying the overall macronutrient ratios by Atwater’s coefficients (14.64 kJ g^−1^ for protein, 35.56 kJ g^−1^ for lipids and 14.64 kJ g^−1^ for carbohydrates^33^).

**Table 5:** Percent macronutrient composition of the major food items in the diet of the red fox (*Vulpes* *vulpes*)^17,31,34,97^.

Food items		Protein	Lipids	Carbohydrates
Wild fruit	*Rubus* sp.	1.3	0.0	5.7
	*Prunus* sp.	1.0	0.2	11.4
	*Sambucus* *nigra*	0.66	0.5	18.4
Cultivated fruit	Plums	0.7	0.28	11.4
	Pears	0.3	0.4	8.9
	*Vitis* *vinifera*	0.5	0.1	13.5
	*Ficus* *carica*	0.75	0.3	19.2
	Undetermined fruit	0.56	0.18	9.6
Insects	Larvae	17.8	13.3	0.0
	Adults	20.3	8.6	0.0
Birds	Anseriformes	18.3	5.95	0.94
	Galliformes	25.8	1.9	0.2
	Passeriformes	21.7	5.4	0.1
	Columbiformes	18.47	23.8	0.0
	Undetermined birds	21.06	9.3	0.3
Mammals	Small mammals	19.6	9.8	0.0
	Lagomorphs	21.8	2.32	0.0
	Ungulates	21.0	0.8	0.0
	*Martes* sp.	20.3	6.9	0.0

To compare the macronutrient composition of the thirty selected diets, we used right‐angled mixture triangles, which represent the three‐component nutritional compositions of diets as Cartesian points in a two‐dimensional nutrient space^30^. Percent protein energy was shown on the third axis (the ‘implicit’, or I‐axis), which varies inversely as distance from the origin increases^30^.

### Study area

**Figure 5:**
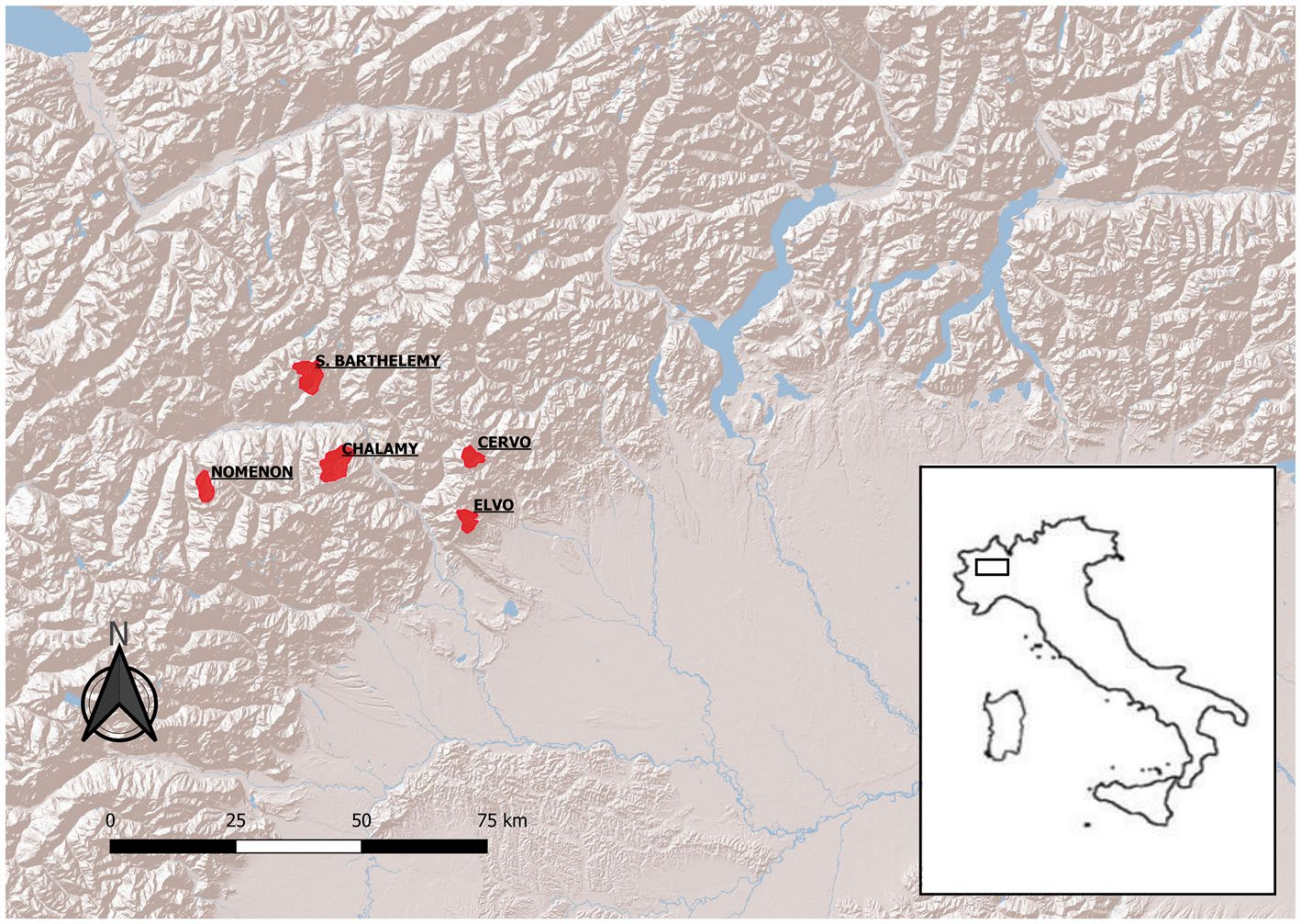
The five study areas (red polygons) in the western Italian Alps where red fox (*Vulpes* *vulpes*) diets, macronutrient ratios and densities were assessed in 2021–2022 (S. Barthelemy: 25.9 km^2^, Nomenon: 14.9 km^2^, Chalamy: 31.4 km^2^, Cervo: 12.3 km^2^, Elvo: 12.1 km^2^). Map created by authors using QGIS 3.14 (https://qgis.org/).

To assess the effect of unbalanced diets on fitness, five areas in the western Italian Alps, ranging between 12.1 and 31.4 km^2^ (Fig. 5), were selected according to the following criteria: (i) altitude ranged between 1000 and 2200 m a.s.l.; (ii) areas had to be well delimited by mountain ridges; (iii) anthropic impact was low, mainly semi-nomadic livestock rearing and slow tourism (hiking, mountain-bike); (iv) hunting pressure, which can alter population density, was negligible.

In general, in all areas the climate is typically Alpine continental with long and cold winters. Snow cover lasts 5–6 months a year with maximum depth during January–February (1.5–2.5 m) and mean temperatures are generally below 0 °C from November to February. Notwithstanding, the two most eastern sampling areas (upper valleys of the rivers Cervo and Elvo, province of Biella, Piedmont) are rainier in May and October–November, while the south-central area (valley of the River Chalamy, Mont Avic Natural Park, Aosta Valley region), is the most xeric.

Between 1000 and 1500 m a.s.l. mixed deciduous woods consist of beech (*Fagus* *sylvatica*), chestnut (*Castanea* *sativa*), ash (*Fraxinus* *excelsior*) and green alder (*Alnus* *viridis*). In the valleys of the rivers Nomenon (Gran Paradiso National Park, Aosta Valley region) and Saint-Barthélemy (Aosta Valley region), above 1500 m coniferous forests predominate, with larch (*Larix* *decidua*), Scots pine (*Pinus* *sylvestris*), Norway spruce (*Picea* *abies*) and silver fir (*Abies* *alba*), which are substituted by mountain pine (*Pinus* *mugo*) in the River Chalamy valley. In the two eastern areas, human activities and climate contributed to prevent the growth of conifers, replaced by shrubs of green alder and hazel (*Corylus* *avellana*). Alpine prairies cover the slopes above 1500 m a.s.l.

### Sampling methods

In each study area, we identified three to five transects (mean length ± SE = 6.2 ± 0.4 km) between 1000 and 2000 m a.s.l. The transects were chosen based on the availability of pathways and were surveyed from March 2021 to March 2022, aiming to collect a minimum of 30 scats per season (October–December: autumn; January–March: winter; April–June: spring; July–September: summer) in each area^55^.

The identification of fox faeces was based on their morphology and size (diameter > 10 mm), which allow to distinguish them from those of other mesocarnivores, such as martens *Martes* spp*.*^56^. Samples were preserved into plastic bags, labelled with an identification number.

Fox numbers were assessed through faecal DNA-based genetic samplings. Between September 2021 and February 2022, we collected 30 samples per area, selecting fresh-looking faeces to obtain amplifiable, non-degraded DNA. Following Ebert et al.^57^, sample size for genetic analysis was calculated as 2.5–3 times the “assumed” number of foxes in each population and based on expected genotyping success (50%, average of previous studies^58–60^). The assumed number of foxes was assessed using available home range sizes for Alpine areas (358 ha)^46^.

For every sample, we withdrew ca. 1 g of faecal material from the external surface, where it is more probable to find flaking cells of the intestinal wall, using disposable sticks (the remaining material was stored for diet analysis). The test-tubes, containing 95% ethanol, were frozen until DNA extraction^61^.

Moreover, fox relative abundance (RA) was expressed as number of scats/100 m of transect^62^.

Sampling was totally non-invasive and did not need the approval of any institutional or licensing committee.

### Diet analysis

We first separated the remains of each prey/food contained in each faecal sample. The minimum number of individuals of each prey type was estimated by the number and position (left/right) of diagnostic hard parts (e.g.: jaw bones for mammals, radio‐ulnae for amphibians). When no diagnostic part was found, the remains of a prey item were considered to belong to a single individual. The relative volume (%V) of each food item “as ingested” was assessed following Kruuk and Parish’s method^60^, which has been widely used for assessing carnivore diets and provides volume estimates as accurate as those obtained by the analysis of stomach contents^40^. The percent frequency (%F) was calculated as the ratio between the number of times (samples) a food item occurs and the total number of analysed scats × 100. The percent mean volume (%Vm = total estimated volume of each food item as ingested/total number of faecal samples = %F × %V/100) reflects the proportional contribution of each food item to the overall diet^63^.

Percent energy ratios were then assessed as so as for literature data and compared using right-angled mixture triangles.

The Chi-squared test (χ^2^) was used to compare the raw frequency data of the major food categories: fruit, insects, birds, mice, dormice, voles, insectivores and ungulates.

To account for multiple tests on related data, the level of significance was corrected using Holm–Bonferroni's sequential technique^64^.

### Genetic analysis

The QIAamp Fast DNA Stool Mini Kit was used to extract the DNA from faecal samples.

We followed the manufacturer instructions, except for final phase, when the ATE buffer was added in three steps of 60 μl each to improve the effectiveness of DNA extraction.

Genotyping was carried out using a multiplex PCR of 20 autosomal microsatellite loci (RF 21, 59, 125, 127, 131, 143, 155, 156, 162, 165, 199, 200^65^; VVM 219, 85, 838, 529, 189, 844, 828^66^), explicitly developed for the red fox.

The quality of DNA was initially screened by four replicated PCRs of two microsatellites. Only those samples showing more than 50% positive PCRs were further amplified four times at each of the remaining 18 microsatellites.

Four multiplex PCRs were conducted, splitting microsatellites based on fragment size and labelling by fluorescent dyes, and using the QIAGEN Multiplex PCR Kit protocol (15 min at 95 °C; 35 cycles of three steps: 30 s at 94 °C, 90 s at 57–63 °C, and 60 s at 72 °C; 30 min at 62 °C; the final volume was reduced to 25 μl).

To lower the probability of retaining false homozygotes or false allele errors, a multitube-approach of 4 independent replicates was used^67^. To construct consensus genotypes heterozygotes were accepted only when the two alleles were recorded in ≥ 2 replicates, while a single allele had to be recorded in ≥ 3 replicates to confirm homozygosity^68,69^.

PCR products were analysed in an automated sequencer ABI 3130XL (Foster City, CA), and visualized using Genemapper (Thermo Fisher Scientific).

### Assessment of population density

To assess the size of the five populations we used CAPWIRE (“CApture WIth REplacement”) estimators^70^, applying the two available models: the Equal Capture Model (ECM), which assumes equal-capture probabilities among individuals; and the Two-Innate Rates Model (TIRM), which assumes that the population includes two groups of individuals, some easy to capture and some that are difficult to capture. The best model was chosen by a likelihood ratio test (LRT) and confidence intervals were estimated through parametric bootstrap.

Population density was calculated as the ratio between population size and the correspondent surveyed area (km^2^). We assumed that mountain ridges coincided with the boundaries of fox home ranges and excluded the steep and rocky areas above 2200 m a.s.l., which were assumed to be not suitable or scarcely used by foxes (surveyed areas: S. Barthelemy: 13.6 km^2^, Nomenon: 11.8 km^2^, Chalamy: 17.4 km^2^, Cervo: 11.3 km^2^, Elvo: 12.1 km^2^).

To assess fox pre-reproductive density, assumed as a rough indicator for fitness, the individuals sampled only once or in autumn, where filed as young of the previous year and discarded.

To assess the relationship between fitness and nutrition, mean deviations of observed ratios from the intake target $\left(\left(\frac{\left|\left(obs-target\right)\right|}{target}\right)\times 100\right)$ were plotted against pre-reproductive density values for each of the five fox populations. The relationships between RA and density and mean deviation of observed macronutrient ratios and pre-reproductive density were tested using linear regression models.

### Supplementary Information

Supplementary Information.

## Data Availability

Data are included in the paper or in the online version as supplementary information. All reviewed datasets are cited in the reference list. For any other reasonable request, contact the corresponding author.
