# Supplementary material for: Nutritional ecology of a prototypical generalist predator, the red fox (Vulpes vulpes)
Source: Sci Rep. 2024 Apr 4;14:7918. doi: 10.1038/s41598-024-58711-6 (PMC10995161; doi:10.1038/s41598-024-58711-6)
Supplement: Supplementary file 1 — Supplementary Information. [file 41598_2024_58711_MOESM1_ESM.docx]

**Nutritional ecology of a prototypical generalist predator, the red fox (*Vulpes vulpes*)**

Balestrieri A.^1^, Gigliotti S.^2,3^, Caniglia R.^4^, Velli E. ^4^, Zambuto F.^5^, De Giorgi E.^3^, Mucci N.^4^, Tremolada P.^1^ and Gazzola A.^3^

^1^Dipartimento di Scienze e Politiche Ambientali, Università di Milano, via Celoria 26, 20133 Milano, Italy.

^2^ Dipartimento di Biologia, Università di Padova, Via Ugo Bassi 58/B, 35131, Padova, Italy.

^3^ Dipartimento di Scienze della Terra e dell’Ambiente, Università di Pavia, I-27100 Pavia, Italy.

^4^Area per la Genetica della Conservazione, Istituto Superiore per la Protezione e la Ricerca Ambientale (ISPRA), via Ca’ Fornacetta 9, 40064 Ozzano Emilia, Bologna, Italy.

^5^ via R. Normanno 179, 92016 Ribera, Agrigento, Italy.

Table SI1. Selected studies for the assessment of fox’s macronutrient intake target.

| **N** | **Reference** | **Study area** | **Latitude** | **Longitude** |
| --- | --- | --- | --- | --- |
| 1 | Balestrieri et al. 2011 | Aosta Valley, NW Italy | 45.44 | 7.19 |
| 2 | Balestrieri et al. 2005 | Valenza, NW Italy | 45.10 | 8.38 |
| 3 | Prigioni et al. 2008 | Val di Fiemme, NE Italy | 46.26 | 11.45 |
| 4 | Remonti et al. 2012 | W Po plain, Italy | 45.25  45.16 | 8.53  9.37 |
| 5 | Goszczynski et al. 1986 | Central Poland | 51.48 | 19.53 |
| 6 | Serafini & Lovari 1993 | Siena, Central Italy | 43.31 | 11.08 |
| 7 | Lucherini et al. 1993 | Maremma National Park, Central Italy | 42.76 | 11.33 |
| 8 | Reynolds & Aebischer 1991 | Dorset, UK | 50.45 | -2.26 |
| 9 | Saunders et al. 1993 | Bristol, UK | 51.27 | -2.35 |
| 10 | Cagnacci et al. 2003 | Gran Paradiso National Park, NW Italy | 45.59 | 7.08 |
| 11 | Sidorovich et al. 2010 | Belarus | 55.02 | 29.02 |
| 12 | Patalano & Lovari 1993 | Abruzzo, Central Italy | 41.41 | 13.50 |
| 13 | Prigioni & Tacchi 1991 | River Ticino, N Italy | 44.51 | 8.75 |
| 14 | Rosa et al. 1991 | N Apennines, Italy | 44.30 | 8.92 |
| 15 | Padial et al. 2002 | Sierra Nevada, Spain | 37.08  41.03 | 3.23  4.53 |
| 16 | Sidorovich et al. 2006 | Belarus | 53.00 | 27.50 |
| 17 | Baltrunaite 2002 | Lithuania | 55.51 | 22.50 |
| 18 | Lanszki et al. 2007 | Hungary | 46.41 | 17.45 |
| 19 | Drygala et al. 2013 | W Pomerania, Germany | 51.00 | 13.00 |
| 20 | Papageorgiou et al. 1988 | Greece | 39.00 | 22.00 |
| 21 | Borkowski 1994 | Poland | 49.26 | 20.00 |
| 22 | Helldin & Danielsson 2007 | S Sweden | 59.40 | 15.30 |
| 23 | Baltrunaite 2001 | E Lithuania | 55.16 | 25.33 |
| 24 | Carvalho & Gomez 2001 | Portugal | 27.25 | -8.17 |
| 25 | Jankowiak et al. 2008 | Wielkopolska province, Poland | 51.34 | 17.40 |
| 26 | Jankowiak & Tryjanowski 2013 |  | 51.34 | 17.40 |
| 27 | Fedriani et al. 1999 | Donana, S Spain | 37.16 | 6.43 |
| 28 | Doncaster et al. 1990 | Oxford, UK | 51.45 | -1.15 |
| 29 | Lanszki et al. 2016 | Hungary | 45.51 | 17.56 |
| 30 | Castaneda et al. 2020 | N France | 48.46 | 2.17 |

Table SI2 Variation (chi-squared test) in the frequency of occurrence of the major food items of the red fox in the five study areas (valleys of the rivers CH-Chalamy, CV-Cervo, EL-Elvo, SB-San Barthelemy and NO-Nomenon).

| **Food items** | **CH** | **SB** | **NO** | **EL** | **CV** | **χ^2^** | **P** |
| --- | --- | --- | --- | --- | --- | --- | --- |
| Fruit | 21.6 | 38.5 | 21.4 | 14.2 | 4.9 | 45.2 | <0.001 |
| Insects | 17.6 | 39.3 | 13.7 | 40.8 | 12.3 | 52.6 | <0.001 |
| Birds | 4.8 | 4.3 | 1.5 | 2.5 | 4.9 | 3.3 | n.s. |
| Mice | 22.4 | 29.9 | 13.7 | 8.3 | 20.5 | 21.5 | <0.001 |
| Dormice | 2.4 | 0.9 | 0.8 | 0.8 | 5.7 | 6.1 | n.s. |
| Voles | 12.8 | 21.4 | 7.6 | 48.3 | 40.2 | 80.0 | <0.001 |
| Shrews | 5.6 | 0.0 | 0.8 | 20.8 | 21.3 | 63.6 | <0.001 |
| Ungulates | 39.2 | 15.4 | 65.6 | 9.2 | 21.3 | 12.1 | <0.001 |


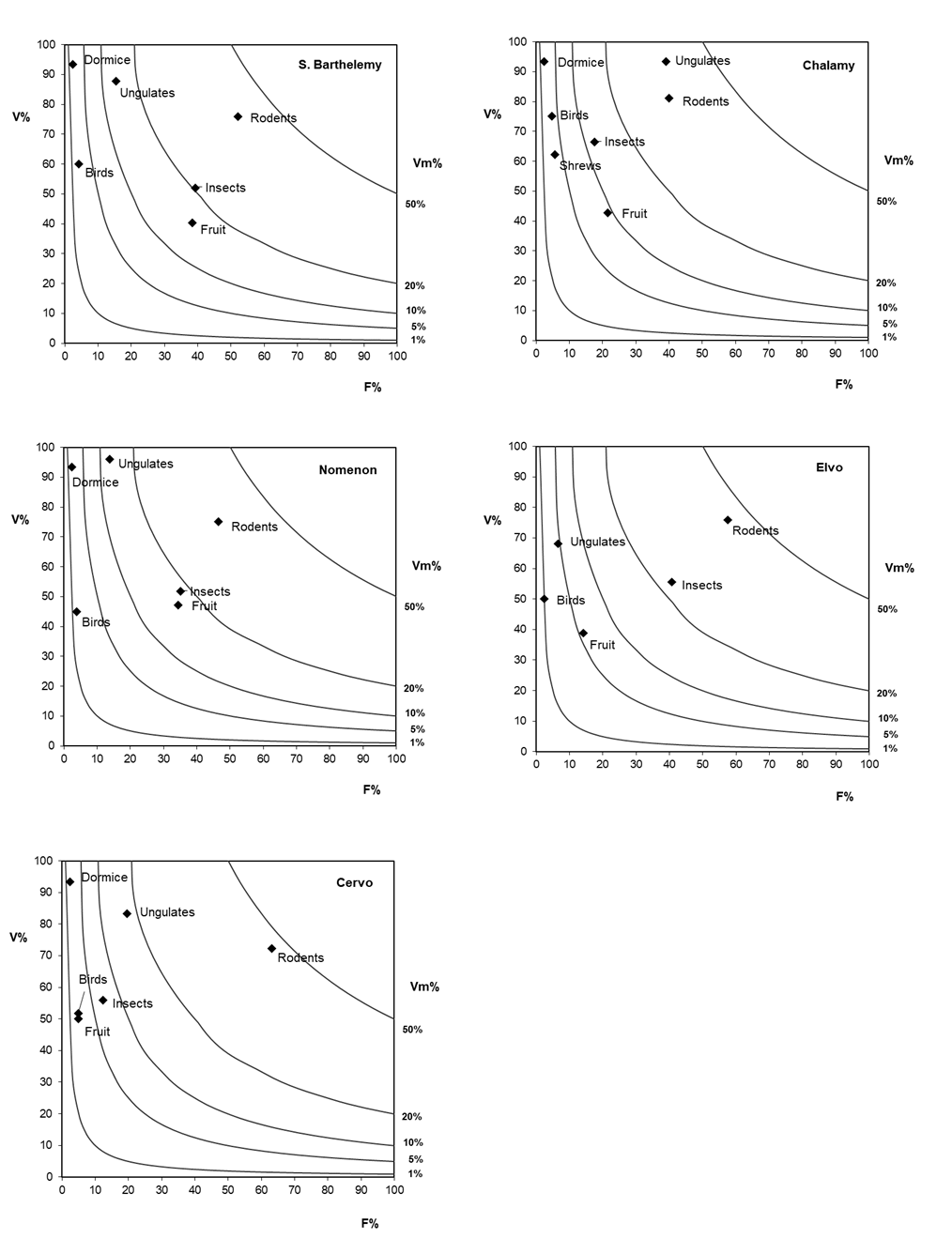


Figure SI1: Estimated volume (%V) of fox main food categories, whenever eaten, vs. their frequency of occurrence (%F) for the five study areas. Isopleths connect points of equal overall mean volume in the diet (Vm%); “Rodents” includes voles and mice.

*
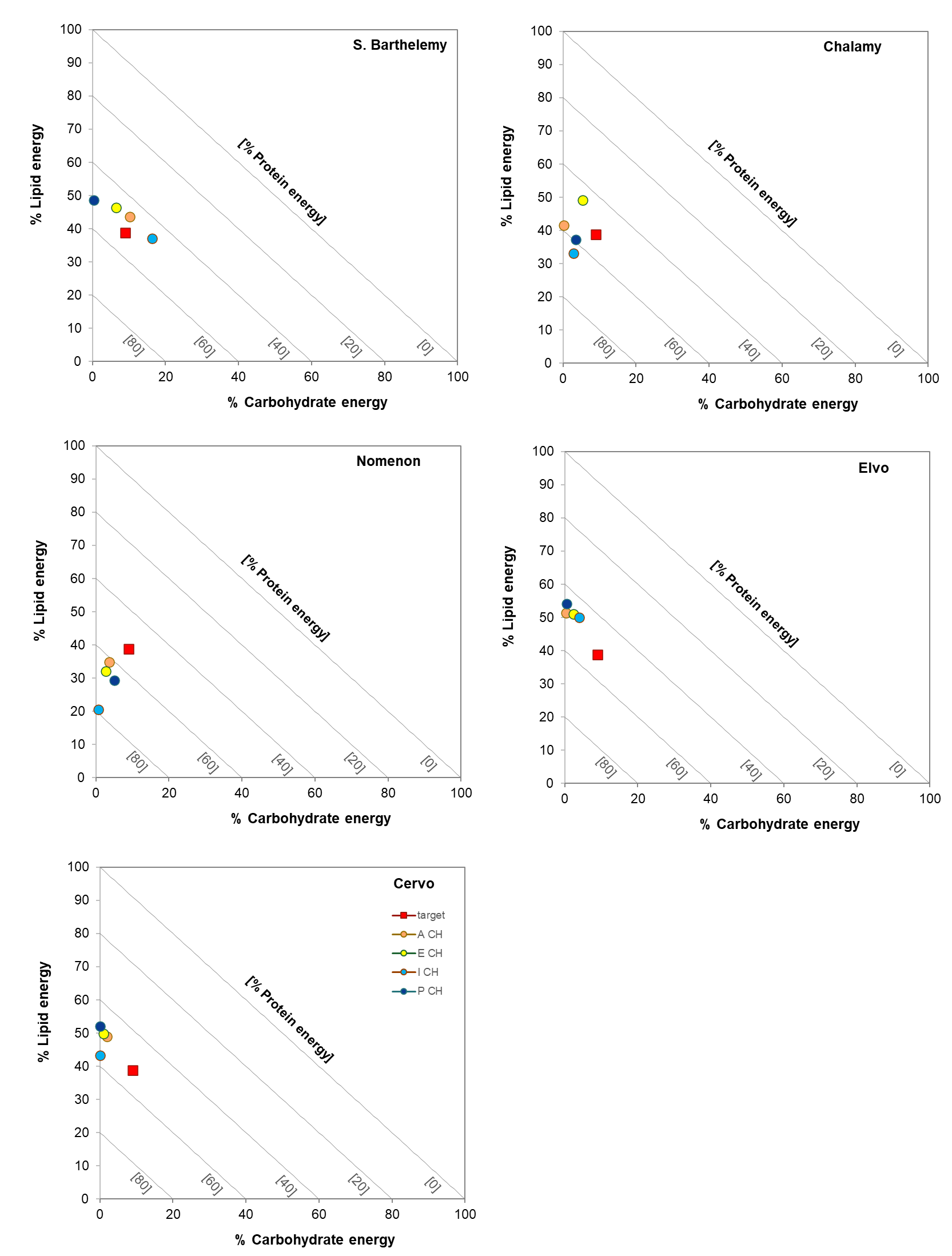
*

Figure SI2. Right-angles mixture triangles showing the seasonal macronutrient ratios for each study area, with respect to the intake target (as assessed by reviewing available literature data).
